# Modeling of Nitrate Leaching during the Fall–Winter Season in Artificially Drained Soils

**DOI:** 10.1100/tsw.2002.204

**Published:** 2002-04-13

**Authors:** Alaa El-Sadek

**Affiliations:** K.U. Leuven, Hydraulics Laboratory, Faculty of Engineering, Kasteelpark Arenberg 40, B-3001 Heverlee, Belgium; National Water Research Center, WRC Building, Delta Barrage, El-Kanater, P.O. Box 13621/5, Cairo, Egypt

**Keywords:** nitrate transport, nitrate leaching, shallow groundwater, fall-winter season, environment

## Abstract

The nitrogen processes that occur within the soil play a major role in determining the nitrate leaching to shallow groundwater. In this study, the transport and fate of nitrate within the soil profile were analyzed by comparing field data with the simulation results of a mathematical model. The objective was to study the transport and fate of nitrate within the soil profile and nitrate leaching to shallow groundwater for the fall-winter season, by applying the methodology in Elverdinge experiment, situated in the sandy loam region in Belgium, from October 1, 2000 to March 31, 2001. The analysis by comparing field data with the simulation results of DRAINMOD-N model is given. The research indicated that the DRAINMOD-N model can, after calibration and validation, be used as a useful fertilizer management tool in predicting the nitrate transport and transformation in the soil profile and the nitrate leaching to shallow groundwater and surface waters. The model can also be used as an environmental control when the environmental objective has a greater importance than profits in the agriculture field.

## INTRODUCTION

In the fall-winter-spring period in Belgium, significant $NO_3$-N losses can occur due to leaching of nitrate that remains in the soil after harvest. Mineralization of organic nitrogen in soil, organic material, plant residue, or manure, in combination with the rainfall excess, increases the leaching of $NO_3$-N during this period. In order to meet the EU-norm of 11.3 mg $NO_3$-N $l^{-1}$ in surface and groundwater, the Flemish Government in Belgium states that the residual mineral nitrogen, measured in the soil profile of cropland (0 to 90 cm), may not exceed 90 kg N $ha^{-1}$ between October 1 and November 15[1]. The value of this residual mineral nitrogen content is still under debate.

The present research project aims to define more accurately the level of mineral nitrogen content in the soil profile that is acceptable in the period October 1 to November 15, and this as a function of soil type, hydrogeological condition, land use, and farming practice. For the analysis, use is made of a mathematical model describing the nitrogen dynamics in agricultural fields. The model used was the DRAINMOD-N model[2]. The advantage of using models is that they facilitate considerably the scenario-analysis in which, for different initial conditions of the residual mineral nitrogen content in the period October 1 to November 15, the leaching can be calculated for various soil types, hydrogeological conditions, land use, and farming practice, and for time series of 30 years. Via statistical interpretation of the simulation results generated in the scenario-analysis, it is feasible to derive the norm for the allowable residual mineral nitrogen content in the soil profile in the period October 1 to November 15, and this as a function of the site conditions.

Before using the DRAINMOD-N model in the scenario-analysis it was calibrated and validated using experimental data of the Hooibeekhoeve, situated in the Kempen (Belgium), and collected in the period 1992 to 1995. Thereafter the calibrated model was used to analyze the transport and fate of nitrate within the soil profile and the nitrate leaching to the shallow groundwater in an experimental field in Elverdinge, situated in the sandy loam region of Belgium, for the fall-winter season (October 1 to March 31). For testing the performance of the model the simulation results were compared to field data. The validation test is called a multisite validation, i.e., the model is calibrated on a given site and the calibrated model is validated to measurements collected on a complete different plot.

## STUDY AREA

Elverdinge is an experimental field situated in Flanders, the northern part of Belgium. Soil physical properties were determined for each soil horizon, using undisturbed core samples. The soil type in the field is classified as a sandy loam soil. The groundwater level fluctuates between 40 and 80 cm below surface. The field was left fallow during the winter period. Soil samples were taken with 3-week intervals in layers of 30 cm, up to a depth of 90 cm. Nitrate concentration of the soil water was determined using suction cups at a depth of 90 cm. Samples of drainage water and groundwater were also analyzed for nitrate. Also, the mineralization rate and denitrification capacity of the soil were measured to get a better estimation of the nitrate leaching.

The soil moisture content was measured at several depths in the soil profile, taking soil samples with an auger in the layers 0 to 30 cm, 30 to 60 cm, and 60 to 90 cm. Taking the wet and dry weight of the soil samples derived the water content. The field is equipped with a subsurface drainage system consisting of parallel, 10-cm diameter, corrugated plastic drains, placed at a depth of 70 cm below surface, and a spacing of 7 m. The soil profile was assumed to have a depth of 4 m, on top of an impermeable layer.

## MATERIALS AND METHODS

The model used for the reconstruction of the nitrogen dynamics in the soil profile was the DRAINMOD model[3]. The model has a drainage component and describes the water movement in the unsaturated zone of the profile root zone using the Richards equation. The simulation output gives the time series of the elevation of the water table and the drain discharge, including the net recharge to the underlying aquifer. DRAINMOD-N is in fact an add-on module to

DRAINMOD, designed for simulating the nitrogen dynamics in artificially drained soils[2,4]. DRAINMOD-N model describes also the nitrogen dynamics in the root zone, using functional relationships. The model is a quasi two-dimensional model because the model considers only vertical transport in the unsaturated zone and both vertical and lateral transport in the saturated zone. The controlling processes considered by the model are rainfall deposition, fertilizer dissolution, net mineralization of organic nitrogen, denitrification, plant uptake, and surface runoff and subsurface drainage losses. Nitrate-nitrogen ($NO_3$-N) is the main N pool considered and the processes in the variable saturated zone of the soil profile can be represented by the advective-dispersive reactive (ADR) equation[2]. Confrontation of the simulation results of the model to a set of experimental field data allows testing the performance of the model.

In the Elverdinge case study, water flow and nitrate leaching are modeled in the flow domain of 3.5-m width, representing half the drain spacing, and a depth of the soil profile of 4 m. The bottom of the soil profile was assumed impermeable. The drain was located at a depth of 70 cm, and was described as a half circular hole with real physical dimensions. The inner wall of the drain was described as a seepage face, implying that the drain was always practically empty. The field data were used to validate the simulation results. The model was tested with respect to its capacity to simulate the fluctuation of the groundwater midway drain spacing, the soil water and nitrate-nitrogen content at different depths in the soil profile, and the nitrate-nitrogen content in the shallow groundwater. Due to limitations in the experimental setup it was not possible to test the model with respect to the drain discharge and the nitrate concentration in the drain water.

## RESULTS AND DISCUSSION

### Calibration and Validation of DRAINMOD and DRAINMOD-N

At the 'Hooibeekhoeve' in the community of Geel (north-eastern part of Belgium) an experimental field trial with maize was set up by the Belgian Soil Service[6] from 1992 to 1995. The soil at the farm site is sandy and classified as a Haplic Podzol, mainly sandy soil with a distinct humus and/or iron B-horizon (a Zdg soil according to the Belgian Soil Classification System). The groundwater level fluctuates between 115 and 160 cm below surface. The first 2 years of the experiment, in 1993 and 1994, maize was sown, whereas in the last season, 1995, the field was left fallow. Different pig slurry fertilizer application packages were applied in spring or autumn. The fertilizer scenarios are listed in Table 1. $NO_3$-N in the fertilizer package is added to the soil solution by dissolution of the fertilizer. Detailed inputs for the maize production practices and $NO_3$-N transport and transformation variables are listed in Table 2. The maize production practices used in the simulations are characteristic for the sandy region of the Kempen[7].

**TABLE 1**
**Field Treatments**

| Treatment | Description of Fertilizer Package |
|---|---|
| 1 | 30 ton $ha^{-1}$ pig slurry applied in autumn |
| 2 | 120 ton $ha^{-1}$ pig slurry applied in autumn |
| 3 | 30 ton $ha^{-1}$ pig slurry applied in spring |
| 4 | 120 ton $ha^{-1}$ pig slurry applied in spring |
| 5 | 60 ton $ha^{-1}$ pig slurry applied in autumn + 60 ton $ha^{-1}$ applied in spring |

**TABLE 2**
**Summary of Inputs for DRAINMOD-N**

| | |
|---|---|
| **Soil properties** | |
| $\theta_{wp}$ (cm$^3$ cm$^{-3}$) | 0.17 |
| Bulk density (g cm$^{-3}$) | 1.6 |
| Organic nitrogen in top soil (μg g$^{-1}$) | 3200 |
| $K_{mnl}$ (day$^{-1}$) | 3.5 x 10$^{-5}$ |
| $K_{den}$ (day$^{-1}$) | 0.01 |
| **Drainage system parameters** | |
| Drain depth (m) | 1.25 |
| Drain spacing (m) | 25 |
| Surface storage (cm) | 2.5 |
| Effective drain radius (cm) | 2.5 |
| **Maize production parameters** | |
| Desired planting date | May 4 |
| Length of growing season (day) | 120 |
| N-fertilizer input (kg N ha$^{-1}$) | 160 |
| Date fertilizer application | May 6, May 14 |
| Depth fertilizer incorporated (cm) | 10 |
| Total dry matter production (kg ha$^{-1}$) | 14500 |
| **Other nitrogen model parameters** | |
| Dispersivity (cm) | 10 |
| $NO_3$-N content of plant (%) | 1.55 |
| $NO_3$-N concentration of rain (mg l$^{-1}$) | 0.8 |

Soil physical properties were determined in one plot of the Hooibeekhoeve (the Kempen, Belgium) for each distinguishable soil horizon, using undisturbed soil samples taken with Kopecky rings. van Genuchten-Mualem parameters for describing the hydraulic functions[8] were fitted on both water retention and multi-step outflow data, using the multi-step outflow program[9]. Basic water retention and hydraulic conductivity curves were established by averaging individual curves for each soil layer. In addition, the soil texture was determined for each soil horizon[10]. The physical properties of the soil are listed in Table 3. The field was intensively monitored during the experimental period. Every 3 weeks, soil samples were taken with an interval of 30 cm to a depth of 120 cm for mineral nitrogen measurements. Mineral nitrogen was measured in groundwater at 200 cm with the same time interval.

During the simulation period the field was cropped with maize. Organic manure was only applied as a fertilizer. Missing data, required to run the model, was either supplementary measured or reconstructed by using the pedo-transfer functions of Vereecken[11], as indicated by Ducheyne and Feyen[10]. The 3-year data (1992 to 1995) was used to extensively calibrate and validate the DRAINMOD/DRAINMOD-N models.

The soil, crop and nitrogen parameters were calibrated resulting in a set of representative parameters for the given soil-crop condition. The calibration of the model parameters was carried out by trial and error[12]. The calibration of DRAINMOD-N model was based on field data of the fertilizer scenario number 3 (30 ton ha$^{-1}$ pig slurry applied in spring); see Table 1. The calibrated model (DRAINMOD-N) was validated vs. data collected on the field fertilizer scenario number 5 and applied to simulate the nitrate transport in the soil profile for the other scenarios (1, 2, and 4).

The statistical results presented in Table 4[13] clearly illustrate that the DRAINMOD-N model performs well in the calibration and validation phase, as well as when the model is used in a predictive mode. The first column in Table 4 indicates that the statistical results of the coefficient of determination ($R^2$) for the linear regression and root mean square residual (RMSR) at 30- to 60-cm depth and bottom of the root zone (90 to 120 cm) are given for calibration

**TABLE 3**
**Soil Physical Properties of the Experimental Field at the Hooibeekhoeve**

| Soil Parameter | Soil Horizon (cm) | | | |
|---|---|---|---|---|
| | **0–35** | **35–50** | **50–100** | **100–200** |
| **Van Genuchten** | | | | |
| θr | 0.055 | 0.019 | 0.011 | 0.017 |
| θs | 0.48 | 0.43 | 0.42 | 0.42 |
| α | 0.016 | 0.028 | 0.032 | 0.019 |
| m | 1.574 | 1.686 | 1.85 | 1.804 |
| n | 0.365 | 0.407 | 0.459 | 0.446 |
| **Mualem** | | | | |
| Ksat, cm day$^{-1}$ | 50.47 | 18.6 | 15.55 | 13.15 |
| λ | 2.031 | 4.041 | 3.314 | 4.007 |
| **Soil texture (%)** | | | | |
| Clay | 2 | 7 | 2 | 2 |
| Silt | 5 | 16 | 3 | 3 |
| Sand | 93 | 77 | 95 | 95 |

**TABLE 4**
**The Coefficient of Determination ($R^2$) for the Linear Regression and Root Mean Square Residual (RMSR) at 30 to 60 cm Depth and Bottom of the Root Zone (90 to 120cm)**

| | 30- to 60-cm Depth | | Bottom of the Root zone | |
|---|---|---|---|---|
| **Scenario** | **RMSR** | **$R^2$** | **RMSR** | **$R^2$** |
| Calibration: Scenario 3 | 0.0568 | 0.74 | 0.0728 | 0.84 |
| Validation: Scenario 5 | 0.0190 | 0.75 | 0.0221 | 0.85 |
| Scenario's analysis | | | | |
| Scenario 1 | 0.0486 | 0.72 | 0.0383 | 0.80 |
| Scenario 2 | 0.0351 | 0.60 | 0.0228 | 0.85 |
| Scenario 4 | 0.0118 | 0.87 | 0.0137 | 0.92 |

(scenario 3), for validation (scenario 5) and for scenario analysis (scenarios 1, 2, and 4), respectively. It is evident that when the tonnage of manure applied increases that the amount of nitrate leaching at the bottom of the root zone increases and exceeds legal limits.

## Modeling of Nitrate Transport and Leaching

To ensure a good modeling of the nitrate leaching, a good water table prediction is a necessity[14]. Therefore, in the first step of the analysis the subsurface drainage discharge and the related groundwater level were modeled for the simulation period October 1, 2000 to March 31, 2001, which is the leaching period in Belgium. Rainfall, evapotranspiration, and subsurface drainage are given in Fig. 1, while the comparison between the simulated and measured water table is given in Fig. 2. Fig. 3 shows the volumetric water content in the soil profile at different depths. Assuming that the water is the vehicle needed to carry nitrate within the soil profile, the results of the water quantity modeling as presented in Figs. 1, 2, and 3 ensure a good nitrate transport and leaching as a second

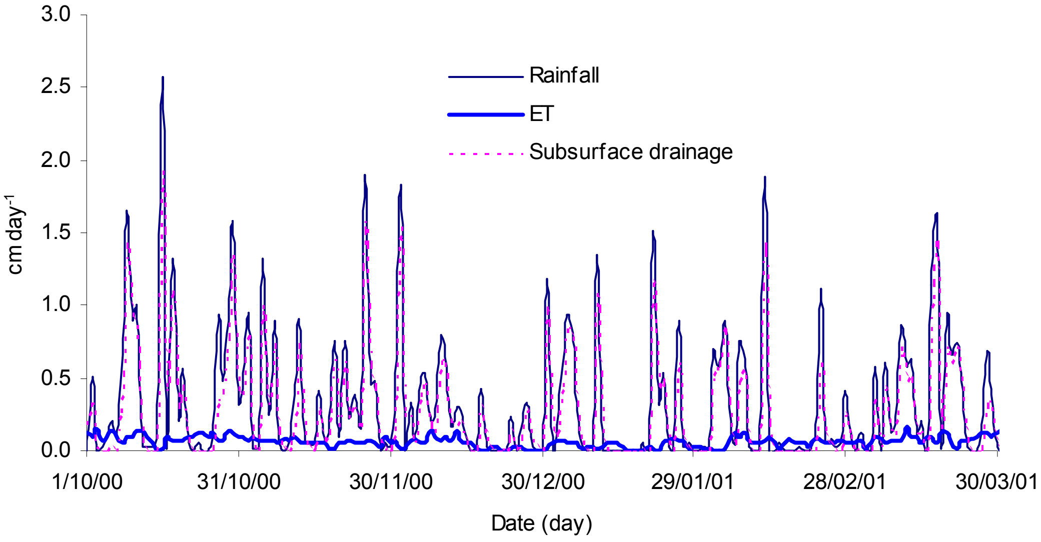

**FIGURE 1**. Rainfall, evapotranspiration, and subsurface drainage in Elverdinge field during the fall-winter season.

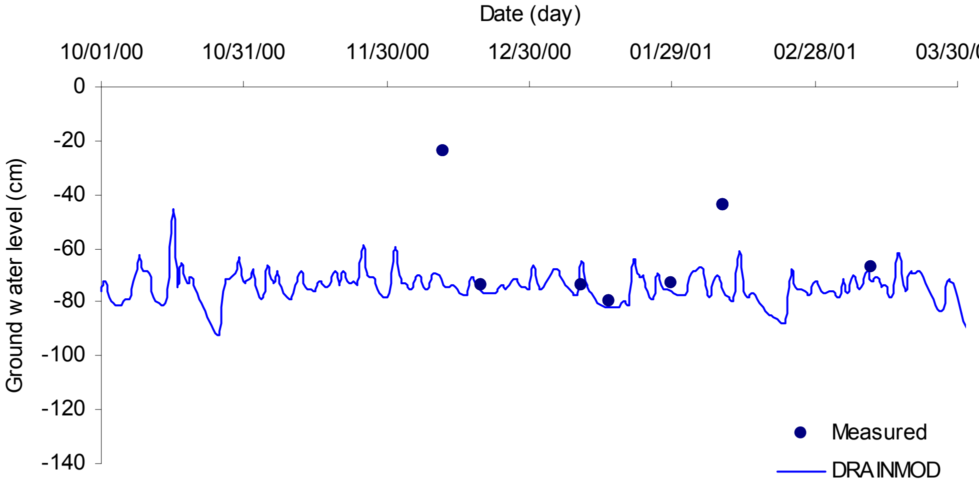

**FIGURE 2**. Simulated and measured groundwater level.

step. During the simulation period, the total amount of rainfall, subsurface drainage and evapotranspiration were 61.3, 50.6, and 11.3 cm, respectively. As expected, there is a high subsurface drainage and a low ET because the simulation period was the fall-winter period.

Due to the surplus of rainfall in the winter season an important part of the nitrate-nitrogen is lost through leaching to the subsurface drainage system. In the simulation period, the amount of $NO_3$-N in the layers 0 to 30 and 30 to 60 cm decreases together with an increase in the layer 60 to 90 cm. Examples of the simulated vs. measured $NO_3$-N concentration (kg $ha^{-1}$) within the soil profile at different depths are shown in Fig. 4. Confrontation of the simulation results to experimental data reveals that the model can simulate acceptably well the water flux and the

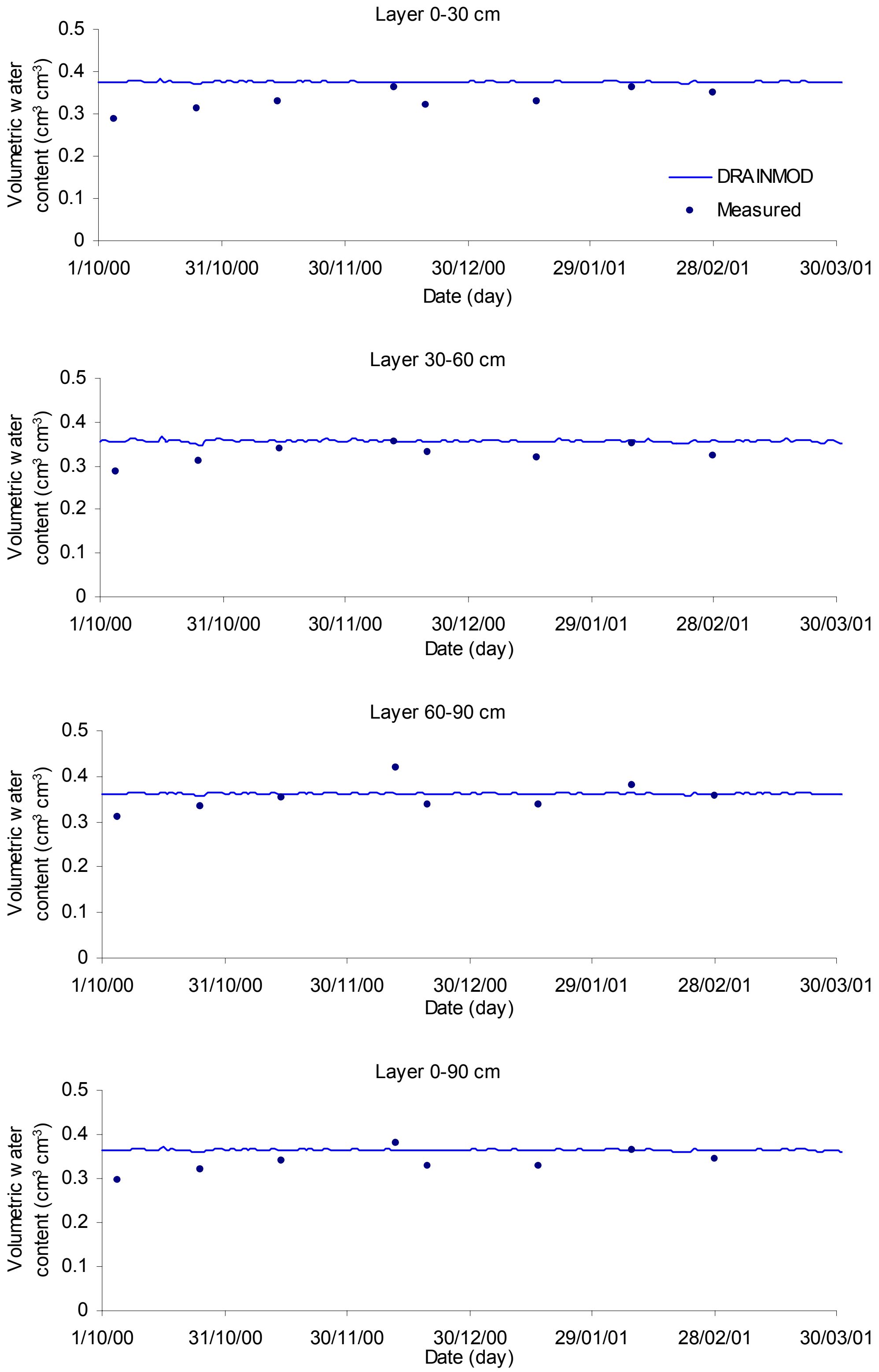

**FIGURE 3**. Simulated and measured volumetric water content at different depths of Elverdinge field.

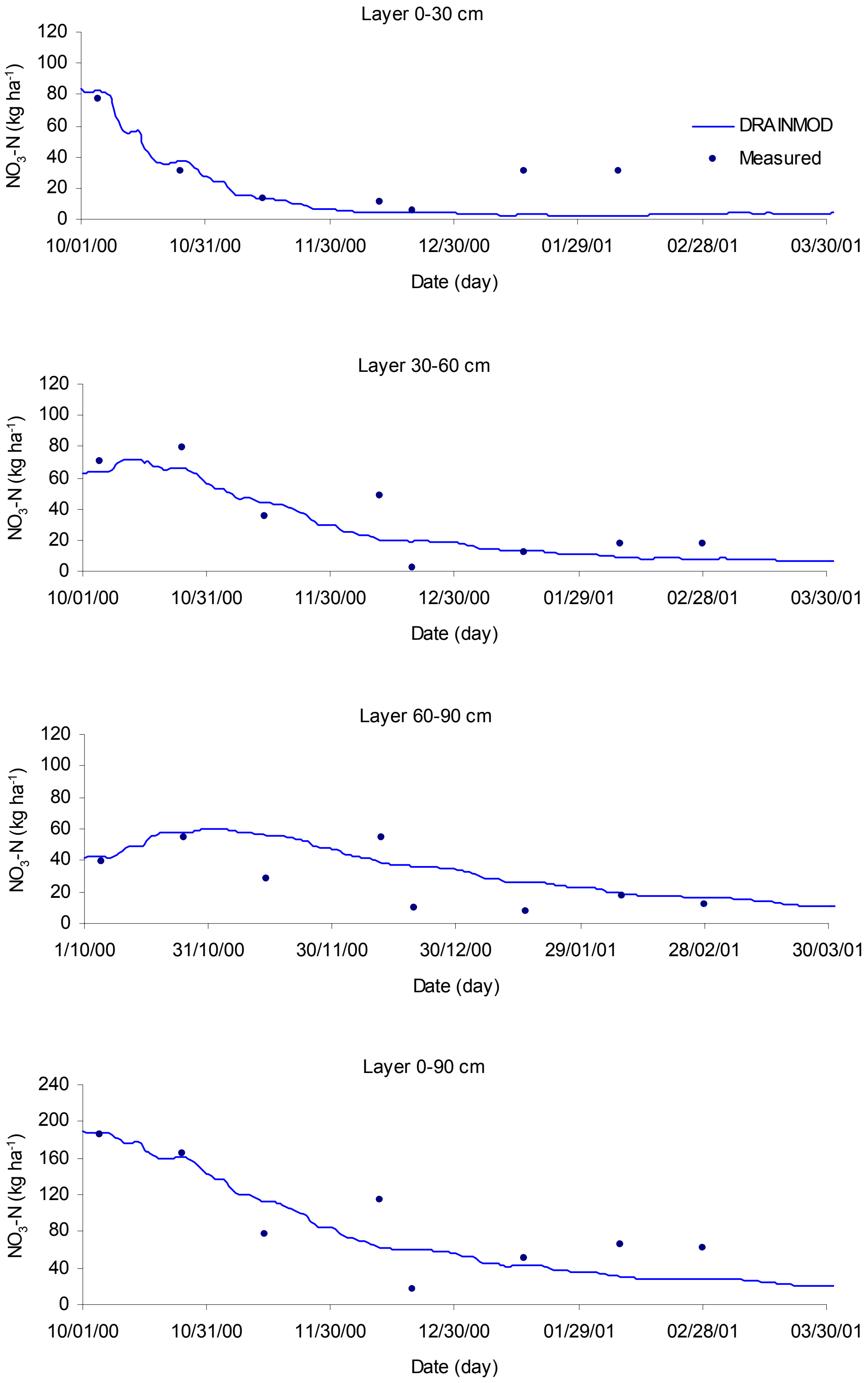

**FIGURE 4**. Nitrate-nitrogen in the soil profile of Elverdinge field at different depths.

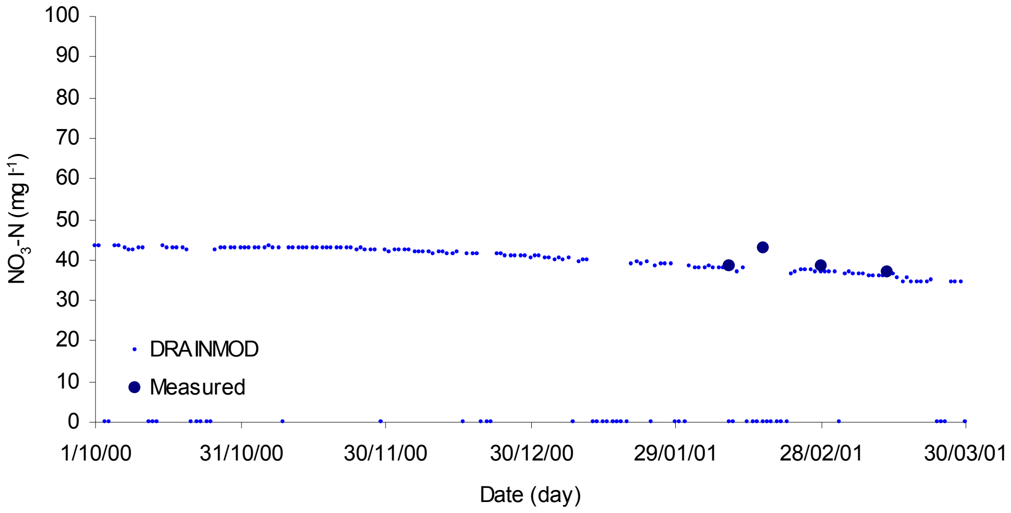

**FIGURE 5**. Simulated and measured nitrate-nitrogen (mg $l^{-1}$) in shallow groundwater during the fall-winter season.

nitrate transport and leaching within the soil profile, provided the model parameters and other input data are available. The model yields good estimates of the nitrate concentrations in the soil. Although DRAINMOD-N is able to predict sufficiently accurate the observed variation of nitrate concentration in the root zone at different depths, the model predicts more accurately the nitrate leaching to drains, resulting in a precise water quality modeling of the nitrate leaching. The nitrate-nitrogen concentration (mg $l^{-1}$) in the shallow groundwater is given in Fig. 5. Although the numbers of the measurements are few points, they match very well with the simulation results.

Fig. 6 depicts the relationship between the rainfall and the nitrate-nitrogen leaching to the field drains. Analysis of the data in this figure reveals clearly that the nitrate-nitrogen leaching to

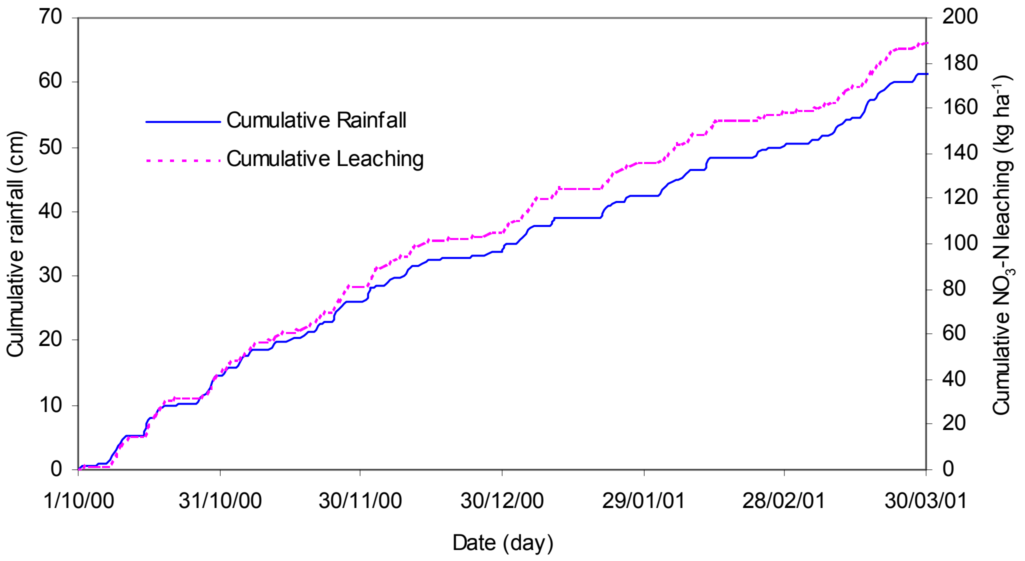

**FIGURE 6**. Effect of rainfall configuration on $NO_3$-N in shallow groundwater.

the shallow groundwater and the related nitrogen processes (mineralization, denitrification, runoff losses, and $NO_3$-N in soil profile) basically depends on the rainfall configuration and amount. The study also revealed that higher amounts of rainfall result in higher nitrate concentration patterns, with more elevated risks if the fertilizer application is applied in autumn.

As was expected, dependent on the high water level (between 40 and 80 cm) during the simulation period (fall-winter season), the denitrification amount is relatively higher than the mineralization amount. Analysis of the simulation results showed that the mineralization rate decreases when the water table is close to the surface and during extended dry periods. $NO_3$-N is depleted from the soil solution by denitrification, drainage, and runoff losses. Denitrification increases during wet periods when the water table is high, but is negligible during dry periods. The simulation results indicated that by increasing the mineralization amount all nitrogen processes (denitrification, plant uptake, runoff losses, subsurface drainage losses, and $NO_3$-N in soil profile) increase. The results also show that by increasing the denitrification process there is a reduction in the amount of $NO_3$-N leached. The study illustrates that on average the DRAINMOD model performs well in the calibration and validation phase, as well as when the model is used in a multisite validation test.

## CONCLUSIONS

The limits for $NO_3$-N concentrations in groundwater and surface waters are still under discussion, but it is likely that they will become stricter. In this study, the DRAINMOD model was used to predict the nitrogen transport and transformation within the soil profile and nitrate-nitrogen leaching to shallow groundwater and surface waters. For the water quantity and quality modeling, climate data of the fall-winter season, collected between October 1, 2000 and March 31, 2001, on an experimental field in Flanders, Belgium, were used. The DRAINMOD model requires limited input. To run the model one needs only time series of daily rainfall, evapotranspiration, maximum and minimum temperature, and few fertilizer application data together with site specific parameters relatively easy to derive. Another important feature of the DRAINMOD model is that it simulates the lateral subsurface flow of water and the $NO_3$-N flux to field drains, ditches, or drains tubes. The analysis indicated that the DRAINMOD model can be used after calibration and validation as a useful fertilizer management tool in predicting the nitrate transport and transformation in the soil profile and the nitrate leaching to shallow groundwater and surface waters. The model can be used to help define a function of the local conditions, the crop cultivated and the climate, the fertilizer package composed of organic and inorganic fertilizers that does not exceed the leaching limits imposed by environmental regulations. Furthermore, the model can be used as an environmental control when the environmental objective has a greater importance than profits in agriculture.

## ACKNOWLEDGEMENTS

This paper was presented at the CSIC/ESF workshop Analysis, Toxicity and Biodegradation of Organic Pollutants in Groundwater from Contaminated Land, Landfills and Sediments, Barcelona, Spain, November 8 to 10, 2001. The author would like to thank professor R.W. Skaggs from North Carolina State University for making available the PC version of the DRAINMOD model, including valuable suggestions for the correct use of the model and proper interpretation of modeling results.
